# Is heart rate response a reliable marker of adenosine-induced coronary hyperemia?

**DOI:** 10.1007/s10554-018-1309-1

**Published:** 2018-02-14

**Authors:** Bhavik N. Modi, Haseeb Rahman, Sara Abou Sherif, Howard Ellis, Kseniia Eruslanova, Amedeo Chiribiri, Divaka Perera

**Affiliations:** NIHR Biomedical Research Centre and British Heart Foundation Centre of Excellence, School of Cardiovascular Medicine and Sciences, St Thomas’ Campus, King’s College London, London, UK

**Keywords:** Adenosine, Hyperemia, Fractional flow reserve, Stress perfusion cardiac MRI, Coronary artery disease

## Abstract

*Introduction* Growing evidence supports ischemia-guided management of chest pain, with invasive and non-invasive tests reliant upon achieving adenosine-induced coronary hyperemia (defined as increased blood flow to an organ’s perfusion bed). In the non-invasive setting, surrogate markers of hyperemia, such as increases in heart rate, are often used, despite not being formally validated. We tested whether heart rate and other non-invasive indices are reliable markers of coronary hyperemia. *Methods* The first part involved Doppler flow-based validation of the best pressure-wire markers of hyperemia in 53 patients. Subsequently, using these validated pressure-derived parameters, 265 pressure-wire traces were analysed to determine whether heart rate and other non-invasive parameters correlated with hyperemia. *Results* In the flow derivation cohort, the best determinant of hyperemia came from having 2 out of 3 of: (1) Ventriculisation of the distal pressure waveform, (2) disappearance of distal dicrotic pressure notch, (3) separation of mean aortic and distal pressures. Within the 244 patients demonstrating hyperemia, non-invasive markers of hyperemia, such as change in heart rate (p = 0.77), blood pressure (p = 0.60) and rate-pressure product (p = 0.86), were poor correlates of coronary hyperemia, with only 37.3% demonstrating a ≥ 10% increase in heart rate that is commonly used to adjudge adenosine-induced hyperemia in the non-invasive setting. *Conclusions* We demonstrate, by correlation with Doppler-flow data, a validated method of identifying coronary hyperemia within the catheter laboratory using the pressure-wire. We subsequently show that non-invasive parameters, such as heart rate change, are poor predictors of coronary hyperemia during stress imaging protocols that rely upon achieving adenosine-induced hyperemia.

## Introduction

A growing body of evidence supports ischemia-guided revascularisation [1]. Surrogates of ischaemia can be assessed non-invasively or during diagnostic angiography, often relying on pharmacological induction of coronary hyperemia [2]. The most widely used invasive measure is fractional flow reserve (FFR) [3–5], based on the measurement of distal coronary and aortic pressure during adenosine-induced hyperemia. Intravenous (IV) adenosine is also used in stress perfusion cardiac magnetic resonance imaging (CMR). This test is increasingly used to detect and quantify ischaemia in patients with suspected coronary disease [6] by demonstrating regional heterogeneity of coronary blood flow during hyperemia. Intravenous adenosine at a dose of 140 mcg/kg/min, has been shown to reliably induce near-maximal hyperemia in most patients, with minimal side-effects [7]. The net effect of IV adenosine in humans is typically a mild reduction in arterial blood pressure associated with increases in heart rate (HR), with multiple mechanisms proposed [7, 8]. Due to its non-selectivity, adenosine also activates other receptors (A_1_, A_2B_ and A_3_), which can also result in cardiac conduction abnormalities, hypotension, flushing and bronchospasm [9].

True hyperemia is best assessed by showing increases in coronary blood flow measured invasively using Doppler or thermodilution techniques, that are difficult to implement outside the research setting. Predicting when a patient is experiencing maximal hyperemia within the catheter laboratory is therefore sometimes assessed by awaiting the onset of flushing, breathlessness and chest tightness symptoms. Additionally, non-invasive surrogates such as blood pressure drop, HR rise and changes in aortic and distal coronary pressure waveforms are relied upon to determine the onset of hyperemia, although no reproducible and objective criteria have been identified.

Within the non-invasive CMR setting, where it is not possible to measure such invasive indices, subjective symptoms along with objective hemodynamic measures of increasing HR and falling systolic blood pressure (SBP) are used as surrogate markers of hyperemia. 10% or 10 beats per minute increase in HR, is commonly considered a marker of adequate hyperemia within the imaging setting, its absence thought to imply inadequate hyperemic stimulus. In these cases, higher adenosine doses are administered or the study is classified as equivocal [10]. A sub-analysis of the CE-MARC study suggests that inadequate hyperemic response is considered a recognized cause of a false-negative CMR perfusion scan [11].

In this study, we tested the hypothesis that HR changes, and other surrogate non-invasive indices are reliable markers of coronary hyperemia.

## Methods

### Study population

Our study population consisted of patients who presented to a single centre for coronary angiography ± proceeding to percutaneous intervention as appropriate. 306 Consecutive patients undergoing FFR measurements between October 2013 and February 2017 were screened, where hyperemia was induced by IV adenosine infusion. Between this period, 53 patients also had simultaneous pressure and Doppler measurements using a CombowireXT guidewire (Philips Volcano) as part of a number of studies utilizing detailed intracoronary physiological measurements in patients with ischemic heart disease. All patients received an IV adenosine infusion dose of 140 mcg/kg/min through an antecubital vein using a standardized infusion pump at a fixed distance from the patient, to minimize variability. For the purposes analysis, FFR was defined as the lowest Pd/Pa ratio following the onset of adenosine, averaged over five cardiac cycles, also known as the ‘smart minimum FFR’ [12]. All participants gave written informed consent in accordance with the protocol approved by the local research ethics committee. The study protocol conformed to the ethical guidelines of the 1975 Declaration of Helsinki.

### Standardisation of invasive pressure assessment of hyperemia

Hyperemia was assessed in the 53 patients using Doppler flow velocity measurements, by examining coronary flow reserve (CFR), defined as the ratio of average peak flow velocity (APV) compared to baseline. Doppler measurements of coronary blood flow velocity have been shown to have inter- and intra-observer variability of approximately 10% [13]. A pre-defined CFR threshold of 1.2 was therefore used to define hyperemia (defined as an increase in blood flow to an organ’s perfusion bed) to ensure that the increase in flow at hyperemia is above the margin of measurement error commonly seen with CombowireXT Doppler flow measurements.

**Fig. 1:**
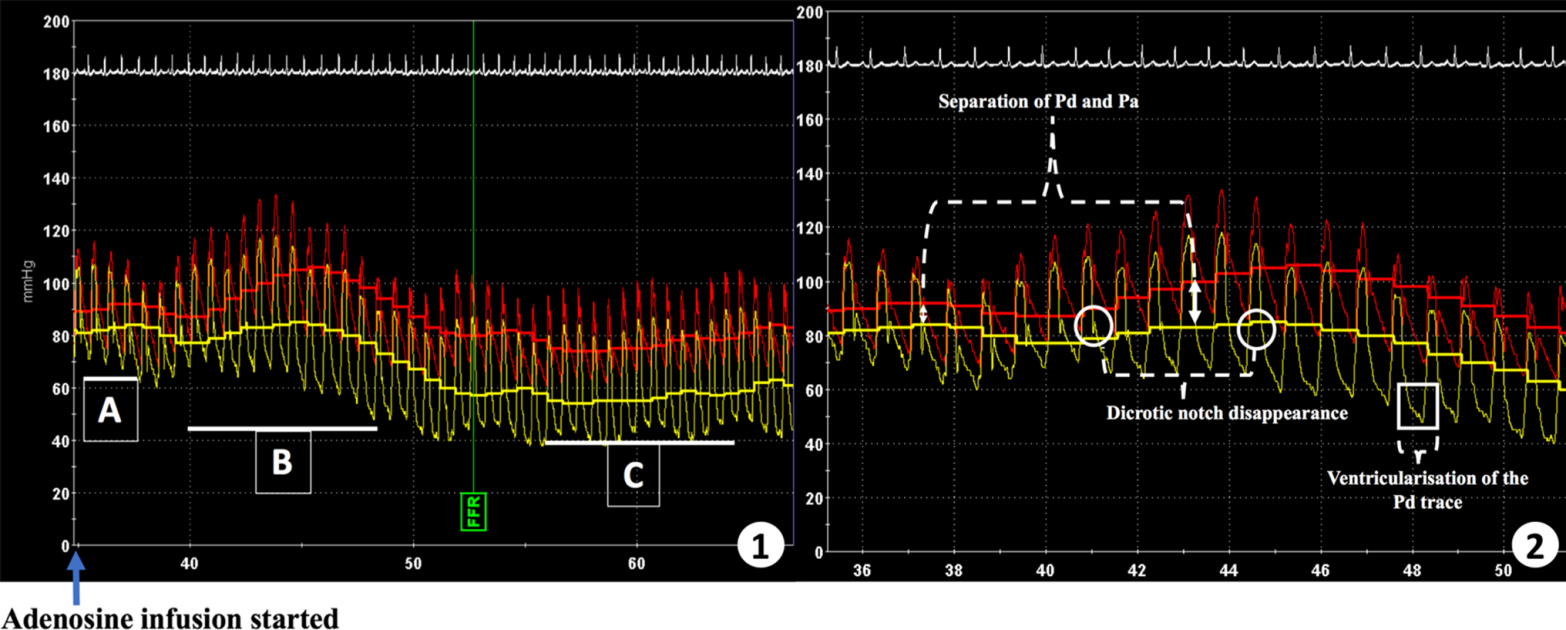
Pressure-Derived Invasive Parameters. **1** The three-invasive pressure-based parameters that were investigated (*A*, *B* and *C*) and subsequently used to define hyperemia during IV adenosine infusion. Red trace = Pa (aortic wave), yellow trace = Pd (distal coronary wave). **2** Magnification of the three-invasive pressure-bounded parameters

In this Doppler cohort, we assessed the diagnostic performance of three commonly-used invasive pressure-waveform parameters of hyperemia, and combinations thereof by calculating their sensitivity, specificity and positive and negative predictive values. These are (1) ventricularisation of distal pressure waveform (a presystolic deflection resembling an ‘a wave’, a slower upstroke of the waveform and a steeper down-stroke than that of aortic pressure [14]), (2) separation of mean aortic and distal coronary pressure [> 10% difference in (Pa–Pd), over five consecutive heart beats, compared to the resting gradient] and (3) disappearance of dicrotic notch from the distal arterial pressure trace (see Fig. 1). In the absence of an established CFR cut-off for defining hyperemia, the diagnostic performance of the pressure-based parameters at CFR of 1.2 were also compared to their performance at a higher CFR of 1.5.

### Assessing adenosine-mediated changes in coronary microvascular resistance and peripheral vascular resistance

The flow cohort enabled characterization of coronary microvasculature resistance (MR) and peripheral vascular resistance (via the augmentation index, AIx) [15], calculated over five consecutive beats at rest and hyperemia. MR was defined as the distal pressure divided by APV. AIx; a measure of central aortic pressure-waveform enhancement by a reflected pulse wave, is calculated as the difference between this late systolic pressure *P*_*2*_ and early systolic pressure *P*_*1*_, as a percentage of pulse pressure, whereby *P*_*1*_ was identified as the first peak on an aortic pressure wave (resulting from the ejection of blood from the heart) and *P*_*2*_ identified as the second peak (resulting from reflection of blood due to constriction downstream in the peripheral vascular tree).

### Assessing diagnostic performance of heart rate and other non-invasive surrogate markers of hyperemia

Based on the diagnostic performance of the invasive pressure parameters (and combinations thereof) in the Doppler cohort, the study population (n = 265) were dichotomously classified as hyperemic or non-hyperemic. The predictive accuracy of commonly used non-invasive haemodynamic markers [HR, SBP, and rate pressure product (RPP, HR × SBP)] were analysed as a percentage change in each parameter in response to IV adenosine-induced hyperemia.

### Statistical analysis

Continuous variables were assessed for normality and if found to be normally distributed, were expressed as mean ± standard deviation. Categorical variables were expressed as counts and percentages. Differences in continuous variables were assessed by an independent Student’s *t* test, whilst differences in categorical variables were evaluated by Fisher’s exact Chi-Squared test. Differences in continuous matched variables, such as HR at rest and at hyperemia were assessed using a Paired *t* test. The diagnostic accuracy of invasive pressure-waveform parameters at detecting a CFR > 1.2 was classified in terms of specificity, sensitivity, positive predictive value (PPV) and negative predictive value (NPV). Changes in microvascular resistance and peripheral vascular resistance were correlated using Pearson’s Rank Correlation, after testing for normality, and results reported a R^2^ values. Statistical analyses were performed using SPSS software (version 22.0; SPSS Inc., Chicago, IL, USA).

## Results

### Performance of invasive pressure-indices of hyperemia

**Fig. 2:**
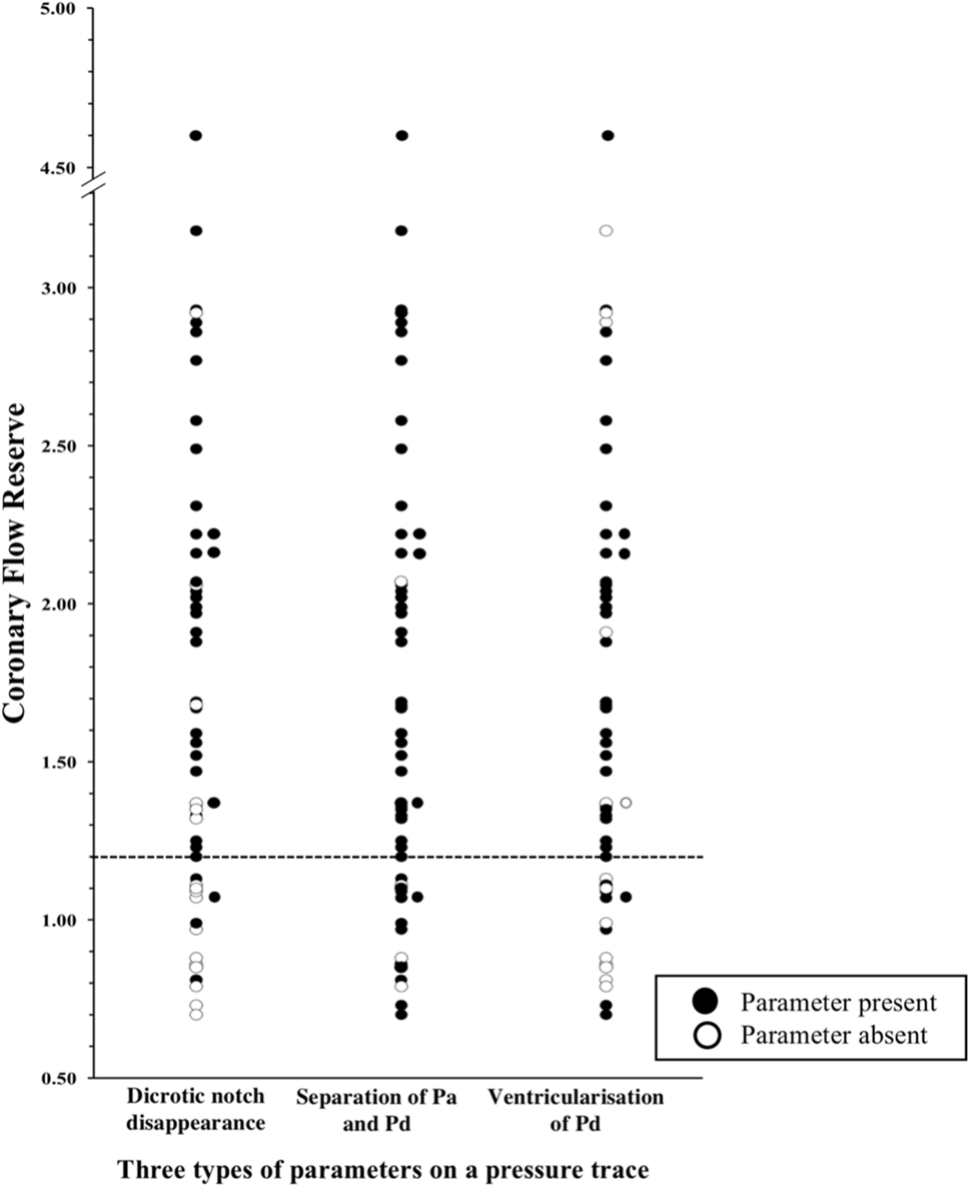
Relationship between coronary flow reserve (CFR) and the three invasive parameters of hyperemia: graph illustrating the presence and absence of pressure-based parameters of hyperemia in 53 patients where simultaneous CFR value were measured. CFR cut-off of 1.2 used as a marker of definitive hyperemia

In the 53 patients with simultaneous pressure and Doppler measurements, mean FFR was 0.84 ± 0.1. The individual pressure-waveform indices all had good sensitivity at detecting hyperemia, defined using a CFR threshold of 1.2, but relatively low specificity. Disappearance of the dicrotic notch and ventricularisation of the distal pressure waveform were the two parameters achieving the best NPV and PPV (Fig. 2; Table 1). The reduced diagnostic performance of Pd and Pa trace separation (Fig. 2), can be explained by the fact that this parameter is difficult to detect in normal coronary arteries (FFR > 0.9). In contrast dicrotic notch disappearance and ventricularisation were detected easily, regardless of disease burden. Combining these indices improved specificity and hence the presence of at least 2/3 pressure indices was chosen as the optimum criterion for detecting hyperemia, forming the basis of detecting hyperemia in the pressure cohort.

**Table 1:** Diagnostic performance of each invasive parameter, and combinations of 2, at a CFR threshold of 1.2

	Sens	Spec	NPV	PPV
Dicrotic notch disappearance	84.2	73.3	88.9	64.7
Separation of Pa and Pd	97.4	20	75.5	75
Ventricularisation of Pd trace	84.2	53.3	82.1	57.1
Dicrotic notch disappearance + ventricularisation	73.7	93.3	96.6	58.3
Dicrotic notch disappearance + separation of Pa and Pd	81.6	73.3	88.6	61.1
Separation of Pa and Pd + ventricularisation	81.6	60	83.8	56.3

Within the validation cohort of patients with pressure and flow data, a CFR cut-off of 1.2 was used to calculate sensitivity (Sens), specificity (Spec), negative predictive (NPV) and positive predictive value (PPV) of the three pressure based parameters. Diagnostic performance was assessed both on their own and/or in different paired combinations

When a higher CFR threshold of 1.5 was used, the diagnostic performance of the pressure-bounded parameters was almost identical to a CFR of 1.2. However, by using a CFR threshold of 1.5, hyperemic and non-hyperemic rates were found to be clinically unrealistic at 53 and 47% respectively.

### Changes in coronary microvascular resistance and peripheral vascular resistance

Using flow data from 53 patients, we found both the MR and AIx significantly dropped from rest to hyperemia (− 37 ± 29.76%, p < 0.001 and − 7.13 ± 55.32%, p = 0.004). There was no correlation between HR change and AIx, R^2^ = 0.031, p = 0.2 (2-sided), or between changes in MR and AIx from rest, R^2^ = 0.021, p = 0.3.

### Dichotomization of patients to hyperemic and non-hyperemic

**Fig. 3:**
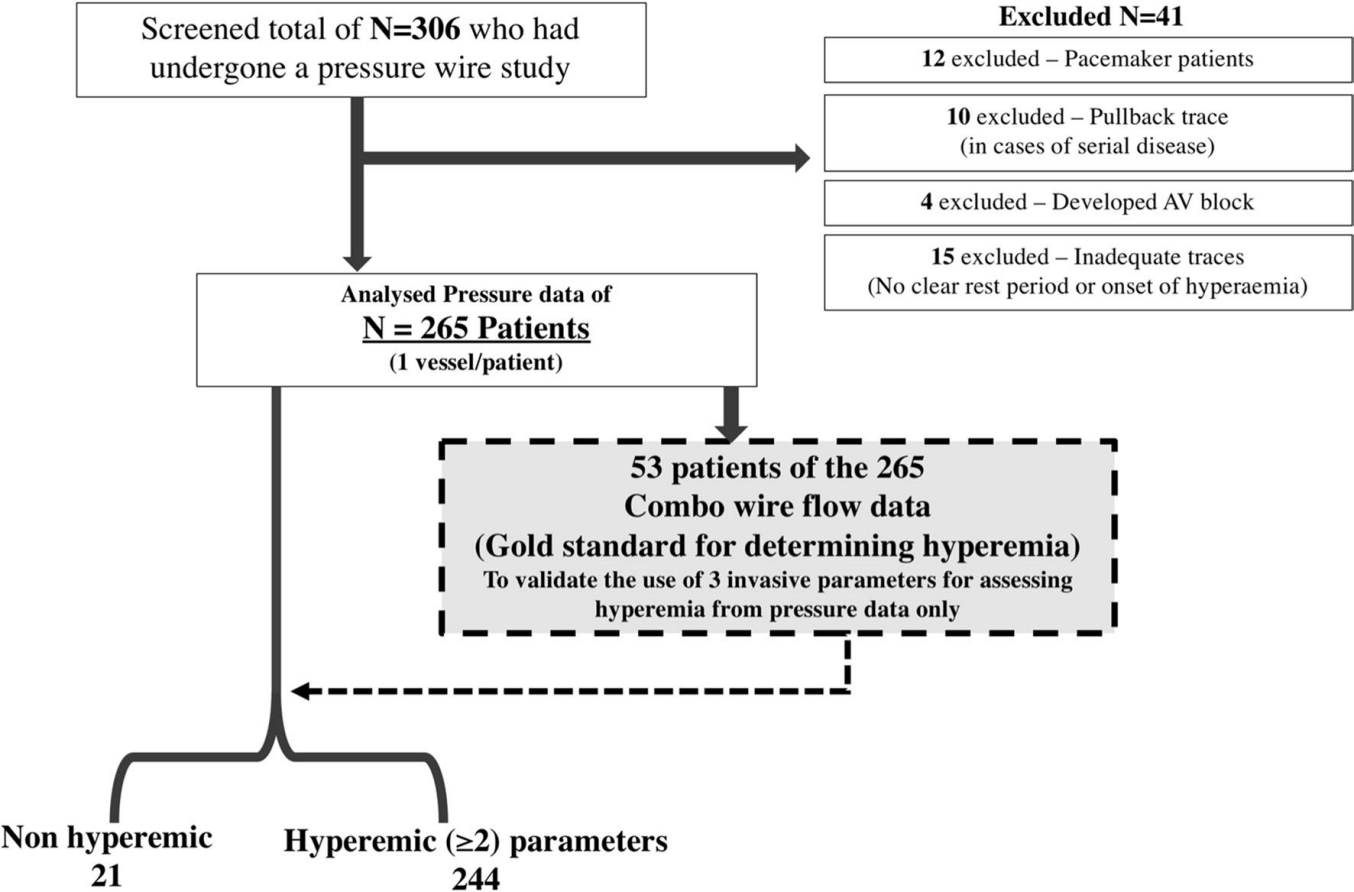
Flow chart: 265 patients with pressure data were analysed using the flow-validated pressure indices to determine hyperemic or not. The predictive accuracy of commonly used non-invasive haemodynamic markers (*HR* heart rate, *SBP* systolic blood pressure and *HR* × *SBP* rate pressure product) were analysed as % change in each parameter in response to IV adenosine from rest to the onset of the lowest Pd/Pa ratio

306 Consecutive patients undergoing invasive FFR measurements between October 2013 and February 2017 were screened, where hyperemia was induced by IV infusion of adenosine. 265 patients were analysed; 41 patients were excluded for reasons specified in the study flow chart (Fig. 3). Based on our validated pressure-based criteria, 244 of the 265 patients were determined to have developed hyperemia.

### Patient characteristics

The enrolled population of 265 patients was 65 ± 11 years old with 74% male. We assessed one vessel per patient and found the mean FFR, in cases where hyperemia was adjudged to have been reached, was 0.81 ± 0.09. Although the proportion of patients with previous PCI was higher patients determined to have reached hyperemia compared to those that did not, there were no other significant differences in patient characteristics between the hyperemic and non-hyperemic groups (Table 2).

**Table 2:** Demographics of patient population

	Hyperemic		Non hyperemic		p value
Variables	N = 244	%	N = 21	%	
Age	65 ± 10.8		67 ± 9.7		0.63
Sex (M/F)	181/63	74.2/25.8	16/5	76.2/23.8	0.54
Hypertension	152	62.3	10	47.6	0.14
Hypercholesterolemia	181	74.2	16	76.2	0.54
Diabetes mellitus	59	24.2	8	38.1	0.13
Smoker	49	20.1	4	19	0.59
Patients with a history of MI	61	25	3	14.3	0.21
Patients with a history of PCI	90	36.9	3	14.3	0.03
Patients with a history of CABG	10	4.1	0	0	
Indication for PCI: stable elective	221	90.6	20	95.2	0.41
Indication for PCI: ACS	23	9.4	1	4.8	0.41

Comparison of demographics in hyperemic and non-hyperemic groups

*M* male, *F* female, *MI* myocardial infarction, *PCI* percutaneous coronary intervention, *CABG* coronary artery bypass graft, *ACS* acute coronary syndrome

*p value calculated by independent samples *t* test for age variable and Chi-squared significance for remaining variables

### Assessment of heart rate and other non-invasive surrogate markers of hyperemia

**Fig. 4:**
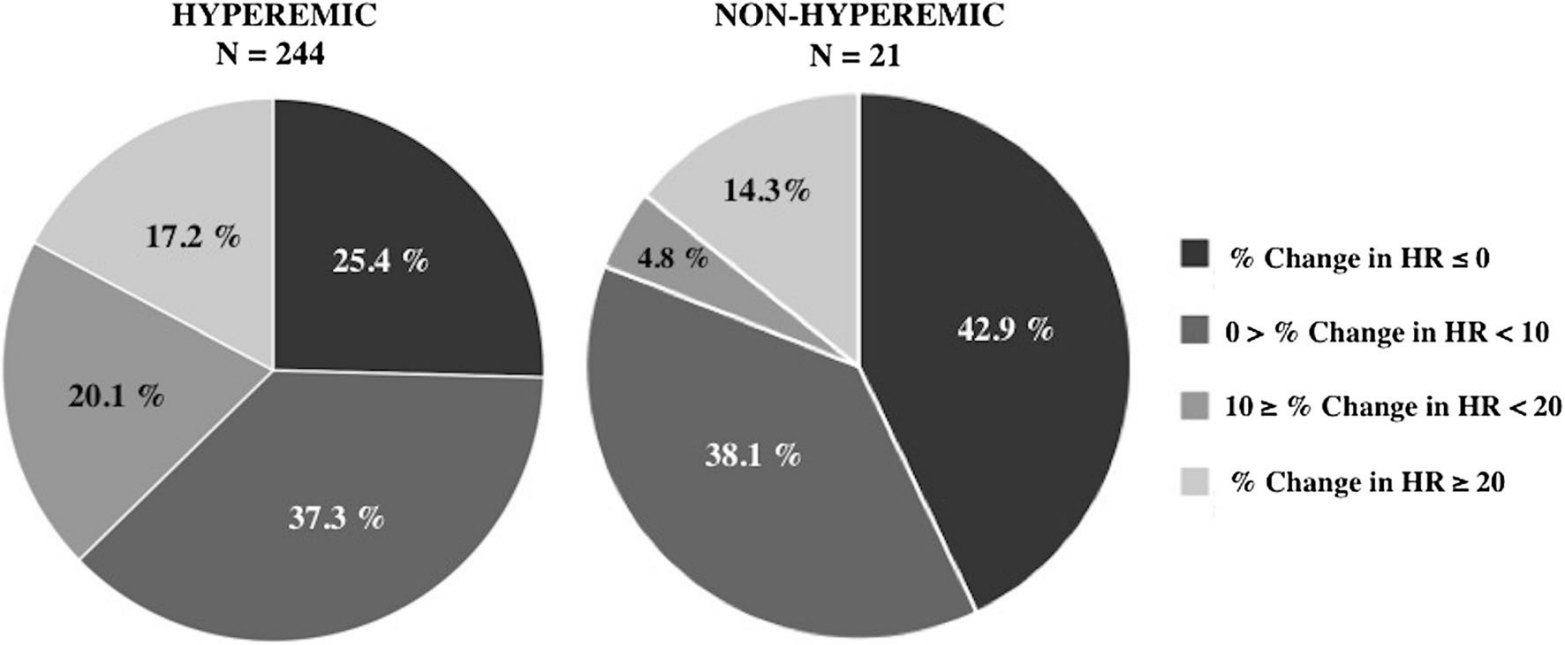
Heart rate variability. Pie chart representation of variability in percentage change in HR in patients deemed to be hyperemic versus those that were not

**Fig. 5:**
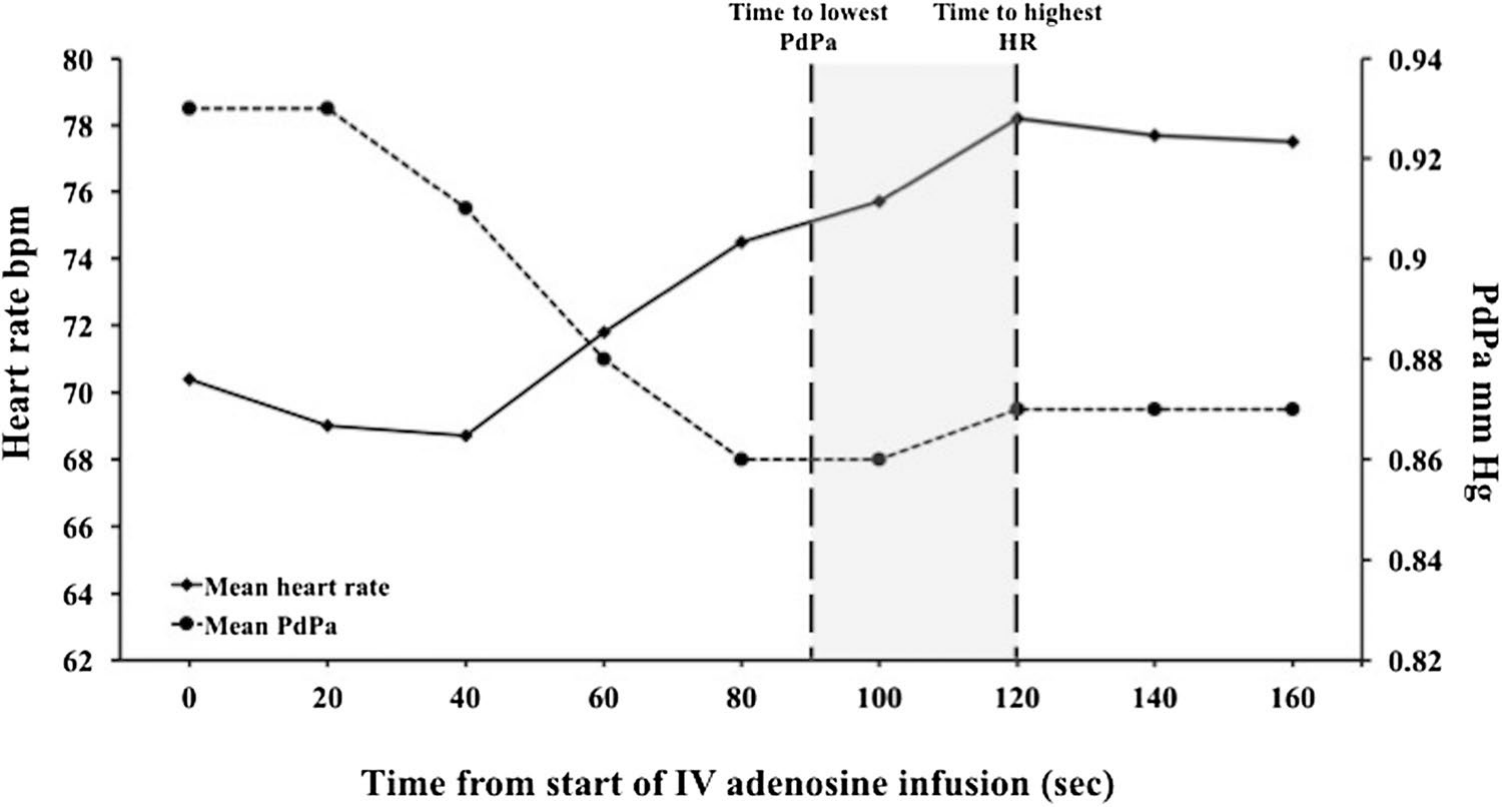
Variability in heart rate and Pd/Pa over time. An illustration of how the mean HR and Pd–Pa changed over the course of adenosine infusion in 244 hyperemic patients

**Fig. 6:**
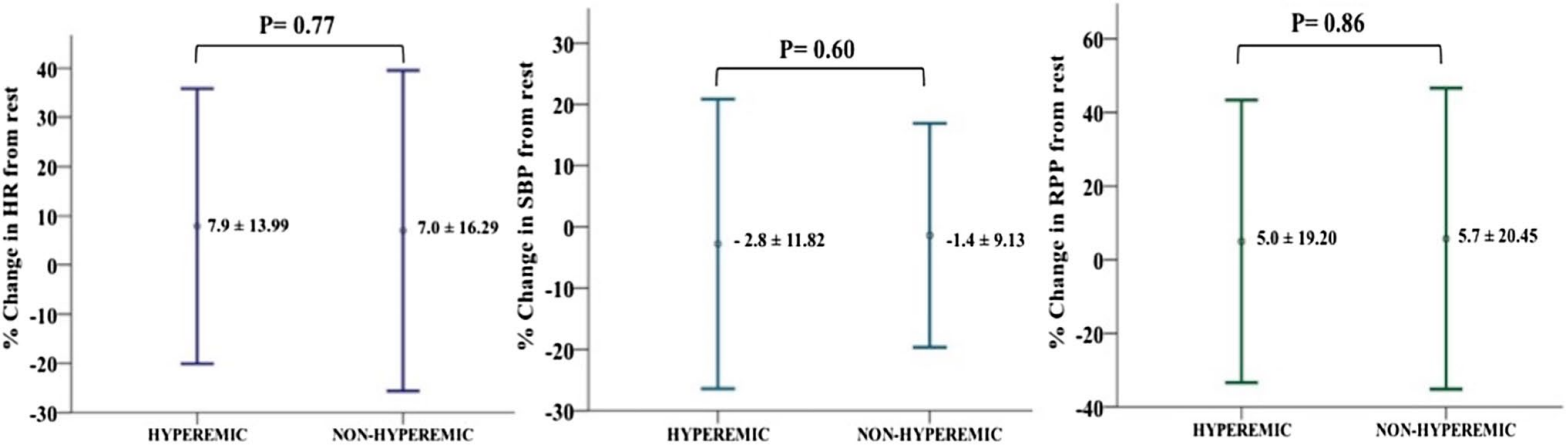
Variability of non-invasive surrogate markers of hyperemia: a comparison of haemodynamic markers (RPP, HR and SBP) between patients achieving hyperemia and those that did not. Values are quoted as means ± standard deviation

The percentage change in HR from rest did not differ significantly between hyperemic and non-hyperemic groups; 7.9 ± 14.0 and 7.0 ± 16.3 respectively (p = 0.78). In addition, there was no significant difference in the proportion of patients exhibiting a ≥ 10% increase in HR between these groups; 37 vs. 34%, p = 0.10 (Fig. 4). Similarly, when these non-invasive parameters were assessed in the 53 flow-data cohort, there was no significant difference in HR, RPP and SBP from rest between hyperemic and non-hyperemic patients, as defined by flow. Of the 38 hyperemic individuals (as defined by CFR ≥ 1.2), only 47% showed a ≥ 10% increase in HR. Overall, assessment of the diagnostic performance of HR in the validation cohort revealed a sensitivity of 37.3%, specificity of 81%, PPV of 96% and NPV of 10%. Similarly, when assessing HR in the flow-cohort, where hyperemia was determined using direct Doppler measurements, HR showed a sensitivity of 47.4%, specificity of 86.7%, PPV of 90% and NPV of 39.4%. In patients where hyperemia was achieved, the lowest Pd/Pa value occurred at 87.8 ± 32.6 s: significantly earlier than the mean time to peak HR (Fig. 5). Other commonly used non-invasive surrogate markers, such as SBP and RPP, also did not vary significantly between hyperemic and non-hyperemic patients (Fig. 6).

## Discussion

In this study, we have demonstrated that the tachycardia associated with IV adenosine infusion is an unreliable surrogate marker of maximal hyperemia, with only 37.3% hyperemic patients exhibiting a 10% increase in HR. This has important implications during non-invasive testing, such as perfusion CMR, when a 10% (or 10 bpm) increase in mean HR is often used as a marker of hyperemia, although there is no consensus method recommended in the guidelines [10, 16].

The first part of this study demonstrated the best performance for assessing hyperemia came from having 2/3 of: (1) Ventriculisation of distal pressure waveform, (2) disappearance of distal dicrotic pressure-waveform notch, (3) separation of aortic and distal mean pressures (see Fig. 2; Table 1). Using ≥ 2/3 of these parameters, we found 8% of patients did not exhibit a hyperemic response. Other studies measuring coronary flow during IV adenosine administration found similar rates of submaximal blood flow [7, 17, 18], however studies that adjudged hyperemia by only demonstrating separation between Pd and Pa traces found higher rates of ‘non-hyperemic response to IV adenosine’ [18]. We demonstrated that this parameter by itself poorly discriminates true hyperemia (Fig. 2), unsurprising in cases of minimal stenoses, where appreciable drops in Pd/Pa, even during hyperemia, may not occur [19]. A CFR of 1.5 was also assessed and whilst similar diagnostic performance was found, the resulting rate of hyperemia was only 52.8% which does not reflect a realistic response to adenosine in the clinical setting, nor does it coincide with the rates of hyperemia reported in previously. Whilst it may be surprising that in the derivation cohort of 53 patients, around half had CFR < 1.5, this group of patients were pre-selected to consist of an intermediate coronary artery disease severity, whereby associated microvascular dysfunction may have contributed to overall reduced flow augmentation following adenosine administration. A CFR cut-off of 1.2 was therefore used to not only compensate for the classical 10–15% error of Doppler measurements, but also to yield clinically realistic PPV, NPV and hyperemia rates, compatible with previous reports [7].

Nearly two-thirds of patients with demonstrable hyperemia did not have an accompanying ≥ 10% HR increase commonly used to infer hyperemia non-invasively. Consequently, the NPV of HR was found to be poor. The poor performance of HR in the flow-defined group further supports its unreliability as a surrogate marker of hyperemia. Had this arbiter been applied in the perfusion MRI setting, these patients would have been misclassified as “non-responders”, potentially leading to unnecessarily higher doses of adenosine (with more unpleasant side-effects) without added diagnostic value or misclassifying negative perfusion scans as equivocal [7, 10, 20]. We have also shown that the time-course of HR change may not reflect the onset of maximal hyperemia (Fig. 5), with prolonged adenosine infusions subjecting patients to unpleasant symptoms unnecessarily. Interestingly, we found 12.3% of patients showed a phasic response to IV adenosine; whilst lower than the 39% previously quoted [12], if first pass perfusion image acquisition occurs during the inter-hyperemic window, the diagnostic value of the scan may be compromised, leading to further diagnostic inaccuracy when using IV adenosine in the non-invasive setting.

### Potential mechanisms underlying variation heart rate response to adenosine

The ability of adenosine to induce tachycardia is ascribed to peripheral vasodilatation, with the assumption that HR change is a direct reflection of peripheral vasodilatation. In addition, both direct and reflex baroreceptor-mediated sympathetic activation are thought to play a role [21, 22]. Our study has shown that there was no correlation between HR, MR and AIx, and therefore highlights the likelihood of other mechanisms, beyond peripheral vasodilatation, by which HR increases in response to adenosine; such as action on the sympathetic nervous system [23]. The lack of correlation between HR and peripheral vasodilatation could also be explained by variable peripheral vasodilatory responses and known variations within adenosine receptor signalling pathways across individuals [23, 24].

Even in the presence of a significant HR increase, mean peak HR and mean lowest Pd/Pa occurred at different times (Fig. 5). If peripheral vasodilation is the major determinant of HR increases, this response may be captured at a different time frame to that of maximal coronary vasodilatation and it could be inferred that complete saturation of the coronary and peripheral vascular beds may be occurring at separate times during adenosine infusion. This is supported by the fact that adenosine receptors have been found to vary in affinity for drugs across different vascular beds [25, 26].

If HR is an unreliable surrogate of hyperemia then what other options exist in non-invasive settings, such as CMR? Our data suggests that SBP or RPP changes are similarly unreliable indices of hyperemia (Fig. 6). Another option is to focus on non-haemodynamic markers of hyperemia such as splenic blood flow attenuation, which can be assessed during a single breath-hold without the need for gadolinium and may be a more reliable marker of coronary hyperemia compared to classical haemodynamic markers such as change in HR or SBP [27]. Another potential method of limiting the HR variability is to consider further investigating vasodilator agents with more selective A2A-receptor action within the non-invasive imaging setting, such as Regadenoson [28].

Our study has demonstrated that assessment of hyperemia in the cardiac catheterisation laboratory should rely on a combination of several invasive pressure-waveform based indices. In the non-invasive CMR setting, we demonstrate that HR and other haemodynamic surrogates are unreliable markers of hyperemia, owing perhaps to variability in dose responses in different vascular beds. Although our study did not specifically evaluate variability in symptoms during adenosine-induced hyperemia, anecdotal variability, e.g. chest tightness, breathlessness and flushing, are also likely to be a result of variability in adenosine receptor responses in different vascular beds. Whilst we appreciate that symptomatic changes are important to assess, due to the retrospective nature of the study, we could not assess these in a standardised way. Perhaps assessment of adenosine-induced symptoms in combination with surrogate non-invasive indices would provide a better method for detecting hyperemia in patients receiving IV adenosine until alternative indices are developed.

### Study limitations

This is a retrospectively analysed, heterogeneous cohort of patients who may have had different levels of pre-medication (including sedation), beta-blockade, tobacco smoking and caffeine intake prior to the catheter laboratory visit, confounding their responses to adenosine. However, at our institute, patients are advised to abstain from caffeine and anti-anginal medication, especially beta-blockers, prior to catheter laboratory tests.

Whilst an increase in coronary blood flow as determined by Doppler (CFR) is the gold standard for assessing hyperemia within the Catheter Laboratory, an absence of flow augmentation does not necessarily imply inadequate hyperemic stimulus. This is partly because CFR is dependent on the both epicardial vessels and microvasculature, hence may be an imperfect measure hyperemia, for example in the context of microvascular coronary disease where it may be impossible to distinguish inadequate hyperemic stimulus from diminished responsiveness. In patients who truly are non-responsive to adenosine, a different stressor such as Dobutamine or physical exercise may be more appropriate.

## Conclusion

An increase in HR has high PPV but poor NPV as a surrogate marker of coronary hyperemia in response to IV adenosine. Even in patients who respond, the time to maximum HR is not always an indicator of maximal hyperemia. In the cardiac catheterisation laboratory, hyperemia is best adjudicated by assessing multiple coronary pressure waveform indices, including dicrotic notch disappearance, ventricularisation of the Pd trace and separation of Pd and Pa values, rather than reliance on one index alone. In the non-invasive setting, the high PPV of HR as a surrogate marker of hyperemia can be helpful, when no other reliable physiologic parameters are available, but should be interpreted cautiously. Further research is needed to develop optimal methods for identifying coronary hyperemia outside the catheter laboratory.
